# Immune Response and Gut Microbiota Shift in the Red Palm Weevil (*Rhynchophorus ferrugineus*) Infected With Entomopathogenic Fungus, *Beauveria bassiana*, Reveal Host‐Pathogen Interactions

**DOI:** 10.1002/arch.70183

**Published:** 2026-06-23

**Authors:** Tzu‐Hao Yang, Fang‐Min Chang, Pin‐Chang Chen, Rameshwor Pudasaini, Hsiao‐Pei Lu, Yu‐Shin Nai

**Affiliations:** ^1^ Department of Entomology National Chung Hsing University Taichung City Taiwan; ^2^ Doctoral Program in Microbial Genomics National Chung Hsing University and Academia Sinica Taichung City Taiwan; ^3^ Agricultural Research Development Program Central State University Wilberforce Ohio USA; ^4^ Department of Biotechnology and Bioindustry Sciences National Cheng Kung University Tainan City Taiwan

**Keywords:** entomopathogenic fungus, gut microbiome, immune‐related genes, microbial control agent, red palm weevil

## Abstract

The red palm weevil (RPW), *Rhynchophorus ferrugineus* (Oliver), is a major pest of palm plants. Entomopathogenic fungi (EPF) are considered promising biocontrol agents against RPW. This study investigated the changes in immune‐related gene expression and gut microbiota of RPW larvae infected with *Beauveria bassiana* (Bb‐NCHU‐155). In infected larvae, fungal genome copy numbers in the midgut and hindgut were lower than those detected in the fat body, suggesting that infection primarily occurs *via* the cuticle rather than the digestive tract. Immune‐related gene expression in the fat body increased steadily from 3 days post‐inoculation (dpi), reflecting a typical host response, whereas digestive tissues exhibited fluctuating patterns. The midgut showed peak induction of *C‐type lysozyme* at 3 dpi and *C‐type lectin* at 6 dpi, while the hindgut displayed the highest expression of *serine protease‐like protein* at 3 dpi. Despite an overall downregulation of immune‐related genes, these results indicate tissue‐specific immune responses. Although *B. bassiana* may not infect hosts through the digestive system, gut microbiota composition differed significantly between infected and control groups, with higher relative abundances of Acetobacteraceae, Lactobacillaceae, and Streptococcaceae in the infected larvae. These shifts co‐varied with the expression of immune‐related genes, such as *defensin* and *C‐type lectin*, suggesting potential functional links between gut microbiota and host immunity. This study provides fundamental insights into the effects of EPF on gut microbiota and immune gene expression in RPW, supporting further research into the complex interactions underlying microbial control.

## Summary

- *Beauveria bassiana* infection induces tissue‐specific immune trade‐offs, activating systemic defense in the fat body while promoting tolerance in the hindgut.
- Fungal infection reshapes the gut microbiota, enriching *Lactobacillaceae* and *Streptococcaceae* despite the fat body being the primary proliferation site.
- A “Microbiota‐Immune Axis” is identified, where specific gut symbionts are strongly correlated with the upregulation of systemic antimicrobial peptides.

## Graphical abstract

This study reveals a “Microbiota‐Immune Axis” in *Beauveria bassiana*‐infected larvae, where gut dysbiosis positively correlates with systemic immunity in the fat body. Meanwhile, the hindgut exhibits immune tolerance, highlighting a tissue‐specific trade‐off between combating the pathogen and preserving the gut microbiome.

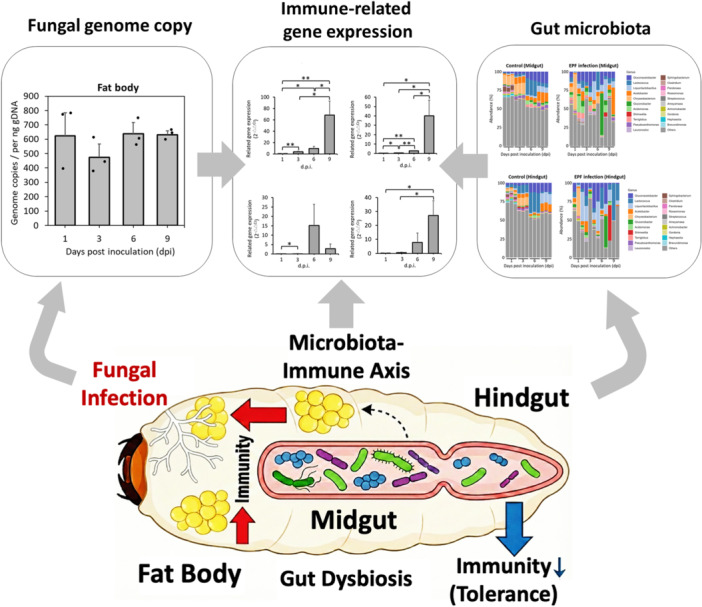

## 1 Introduction

Red palm weevil (RPW), *Rhynchophorus ferrugineus* (Olivier 1790) is a major pest of palm trees and causes economic losses (MacLeod and Hussein 2017). Since its rapid spread during the 1980s (Hussain et al. 2013), RPW has become distributed in 54 countries, including Taiwan. To date, the RPW has been reported infesting 32 species of palm trees (Soroker and Colazza 2017). However, the control of RPW remains challenging due to the concealed habitat of its larvae. While chemical insecticides such as phosphine (Wakil et al. 2018) and ethion (Hussain et al. 2019) are the primary control method, their frequent use may lead to the development of resistance (Al‐Ayedh et al. 2016; Wakil et al. 2018). Therefore, entomopathogenic fungi (EPF), which are eco‐friendly, harmless to non‐target organisms, pose a low risk of resistance, and are easy to mass‐produce, could be a potential control agent for RPW (Hussain et al. 2016; Yang et al. 2023). Among EPF, the use of *Beauveria bassiana* and *Metarhizium anisopliae* for the control of RPW has been widely studied (Sutanto et al. 2021; Yang et al. 2023; Yasin et al. 2019). However, the effectiveness of EPF in controlling RPW may be limited by several factors, such as the immune responses and gut microbiota of RPW (Liu et al. 2021; Muhammad et al. 2019).

EPF infects insects by attaching and penetrating their bodies, subsequently growing extensively and causing death. Upon pathogen invasion, insects trigger immune responses in which pathogen‐associated molecular patterns (PAMPs) bind to pattern recognition receptors, thereby activating downstream immune pathways (Lu et al. 2020). In insects, the fat body serves as the primary immune organ and produces various antimicrobial peptides (AMPs) in response to specific pathogens (Qu and Wang 2018). It was reported that several immunity‐related genes were significantly upregulated in RPW larvae infected with EPF within 24 h (A. Hussain et al. 2016). These genes include pathogen recognition receptors (*C‐type lectin* and *endo‐beta‐1,4‐glucanse*), signal modulators (*serine protease‐like protein*), signal transducers (*calmodulin‐like protein* and *EF‐hand domain‐containing protein*) and immune effectors (*C‐type lysozyme*, *cathepsin L*, *defensin‐like protein*, *serine carboxypeptidase*, and *thaumatin‐like protein*), suggesting their involvement in the immune response against EPF.

Another challenge in controlling RPW with EPF is the role of the gut microbiota. These microbial communities perform multiple functions, including detoxification of plant secondary compounds and degradation of plant biomass, which can improve host nutritional status and physiological condition. This, in turn, may enhance host defenses and reduce susceptibility to EPF infection (Salem and Kaltenpoth 2022). Recently, culture‐dependent and sequencing‐based culture‐independent methods have been applied to investigate the gut microbiota of RPW (Jia et al. 2013; Montagna et al. 2015; Muhammad et al. 2017; Tagliavia et al. 2014). These studies indicated that the gut microbiota of RPW may also enhance host immune responses against bacterial pathogens (Muhammad et al. 2019). However, the roles of gut microbiota in RPW during EPF infection remain unclear.

In this study, a highly virulent EPF isolate, *B. bassiana* NCHU‐155 (Bb‐NCHU‐155), was isolated from a field‐collected RPW pupa and selected for further investigation. The genome copy number of Bb‐NCHU‐155 in infected RPW was quantified, and the relationship between the host immune system and gut microbiota following EPF infection was investigated. In addition, the correlation between gut microbiota and the expression of immune‐related genes was analyzed. We hypothesize that infection by *B. bassiana* may modulate host immune responses and alter the gut microbiota composition of RPW larvae. Furthermore, we propose that shifts in gut microbiota are associated with changes in the expression of immune‐related genes, thereby influencing the host response to EPF infection. The findings of this study will provide valuable insights for improving microbial control strategies against RPW.

## 2 Methods and Materials

### 2.1 RPWs Collection and Laboratory Rearing

The RPW larvae, pupae, and adults were collected from infested Canary Island date palm in Changhua County (multiple sites in Tianwei and Beidou; see coordinates in Table S1) and on the campus of National Pingtung University of Science and Technology (22°38′37.3″ N, 120°36′35.1″ E). The infested date palms were felled using a chainsaw, and the trunks were subsequently cut into smaller sections with a hand saw. These sections were then carefully inspected to locate and collect the larvae, pupae, and adults of RPW. The rearing of RPW was modified from our previous study (Yang et al. 2023). The collected adults were reared in plastic boxes (length = 14.5 cm, width = 10.5 cm, height = 8.5 cm) at 25°C, 65% relative humidity (RH), and a photoperiod of 0:24 (light: dark). Shredded sugarcane pieces (~5 cm) were provided as both for food and oviposition substrates. Newly hatched larvae were reared in plastic cups (diameter = 8 cm, height = 6 cm), under the same conditions as adults. Larvae were fed with cottonwood supplied with 20% honey for the diet and shredded sugarcane pieces (~2 cm). All the individuals used in this study were the offspring of field‐collected individuals.

### 2.2 Isolation and Selection of EPF Against RPW

The EPFs were isolated from the field‐collected RPW cadavers with following a previously described method (Chang et al. 2021; Liu et al. 2021). Briefly, the fungi were isolated from the mycosis RPW using sterilized toothpicks and cultured on quarter‐strength Sabouraud dextrose agar medium (¼ SDA) at 25°C for 7–10 days. The cultured fungi were re‐isolated on ¼ SDA for 2–3 rounds (7–10 days cultured/per round) at 25°C, and then the pathogenicity screening against mealworms (*Tenebrio molitor*) was performed to obtain EPF isolations (Chang et al. 2021; Liu et al. 2021). The isolated EPFs were subjected to pathogenicity screening against RPW. The EPF isolates with high virulence against mealworms were used in the pathogenicity screening against RPW. All EPFs were cultured on ¼ SDA at 25°C for 10–14 days. The conidia were harvested with 0.03% Silwet L‐77 solution, and 10^7^ conidia/mL conidia suspension was prepared. A 10 μl of the conidia suspension was cultured on ¼ SDA on 60 mm petri dishes at 25°C for 10 days. The pathogenicity screening was following by our previous study (Yang et al. 2023). Each of the RPW larva was individually exposed to the fungal cultures by direct attachment to the plate for 5 min, with each fungal isolate tested in three replicates. The mortality of inoculated RPW larvae was recorded daily for 14 days. The fungal mycosis of inoculated RPW larvae was observed daily.

### 2.3 Molecular Identification of EPF

All the EPF isolates showed pathogenicity against mealworms were subjected to molecular identification based on the internal transcribed spacer (ITS) region to determine the fungal genus. Fungal genomic DNA (gDNA) was extracted by the Yeast Genomic DNA Kit (Geneaid, TW) according to the user's manual. The ITS region (ITS1‐5.8S‐ITS2) was amplified by polymerase chain reaction (PCR) using the primer set ITS1F/ITS4R (Table S2). Among these EPF isolates, the isolate that exhibited the fastest RPW‐killing activity was further identified to the species level by amplifying the nuclear intergenic region B locus (*bloc* region) with the primer set Bloc_B5.1F/Bloc_B3.1 R (Table S2). Both of the PCR protocols followed the previously described methods (Wu et al. 2022). All the PCR products were sequenced by a sequencing service company (BIOTOOLS, TW), and the obtained sequences were analyzed by NCBI BLAST to identify the closely related fungal species. The *bloc* sequences of the selected EPF isolate along with those from other closely related EPF strains, which were obtained from the NCBI GenBank, were subjected to phylogenetic analysis (Supplementary Table 2). The sequences were aligned using ClustalX (Thompson 1997). The conserved sequences were manually trimmed with GeneDoc (Nicholas and Nicholas 1997) and then subjected to construct the *bloc* phylogenetic trees by MEGA7 (Kumar et al. 2016) based on the maximum likelihood (ML), neighbor‐joining (NJ), and minimum evolution (ME) models, and the evolutionary distances were computed by the Tamura 3‐parameter method (Tamura 1992). Bootstrap analyses were performed to evaluate the robustness of the phylogenies using 1000 replicates.

### 2.4 Lethal Time (LT_50_) Bioassay

Based on the results of EPFs pathogenicity screening against RPW, the isolate with the fastest RPW‐killing activity was selected to perform the lethal time (LT_50_) bioassay tests. A conidial suspension concentration with 10^7^ conidia/mL was prepared after the EPF isolate was incubated on ¼ SDA at 25°C for 7 days. Fifteen of 8th instar RPW larvae were immersed in 12.5 mL of conidia suspension for 5 min as one replicate. A total of three replicates were conducted in this experiment. For the control group, 12.5 mL of 0.03% Silwet L‐77 solution was used. The mortality of infected RPW larvae was recorded daily for 14 days. The LT_50_ of EPF‐inoculated RPW larvae was calculated by probit analysis with SAS 9.4.

### 2.5 DNA and RNA Extractions of EPF‐Infected RPW Tissues

To better understand the impact of EPF on the RPW larvae, the 8th instar larvae of RPW were infected with Bb‐NCHU‐155 at a concentration of 10^7^ conidia/mL for gut microbiome analysis and immune gene expression. Five RPW larvae per group were immersed for 5 min in 12.5 mL of either conidia suspension or 0.03% Silwet L‐77 solution, serving as the infected and control groups, respectively. For the sampling, three alive individuals from each group were collected at 1, 3, 6, and 9 days post‐infected (dpi), respectively. Prior to tissue dissection of RPW larvae, all samples were immersed in 75% ethanol for 90 s, followed by rinsing with sterilized ddH_2_O, and then the midguts, hindguts, and fat bodies of RPW larvae were dissected by 75% ethanol sterilized scissor, tweezers, and pins. The dissected tissues were transferred to 1.5 mL microcentrifuge tube for DNA and RNA extraction.

DNA and RNA from dissected RPW tissue samples were extracted by Presto DNA/RNA Kit (Geneaid, TW). Briefly, tissue samples were collected in a 1.5 mL microcentrifuge tubes and kept on ice. 400 μL of DR buffer and 4 μL of ß‐mercaptoethanol were added to the sample and homogenized by a sterilized grinding pestle. The homogenized sample was incubated at room temperature for 5 min and then centrifuged at 16,000*g* for 2 min. The sample was transferred into GD columns with 2 mL collection tubes and centrifuged at 16,000 *g* for 1 min. The GD columns were transferred into new collection tubes for DNA purification, and the flow‐through was subjected to RNA purification according to the user's manual.

### 2.6 Detection of EPF Genome Copies

The EPF genome copy number in infected RPW was determined via absolute quantitative PCR (qPCR) using the *beta‐tubulin* Forward and Reverse primer set (Table S2). The qPCR reaction mixture consisted of 10 μL of SensiFAST SYBR Hi‐ROX Kit (Meridian Bioscience, USA), 1 μL of gDNA sample, 1 μL of each primer (10 μM), and 7 μL of ddH_2_O. Real‐time qPCR conditions were as follows: 95°C for 2 min, followed by 40 cycles of 95°C for 10 s and 60°C for 30 s. A standard curve was constructed using genomic DNA extracted from a known concentration of *B. bassiana* conidia (1 × 10^7^ conidia/mL), which was serially diluted 10‐fold down to an equivalent concentration of 1 × 10^5^ conidia/mL. *C*_t_ values were plotted against log_10_ of the initial conidia concentration to generate a linear regression equation: *y* = −3.2105 × + 41.716 (*R* ^2^ = 0.9974) with an amplification efficiency of 104.9%. This standard curve was used to estimate fungal abundance in the tissue samples, expressed as conidial equivalents.

### 2.7 Detection of Immunity‐Related Gene Expression

There are four immunity‐related genes, including *C‐type lectin, serine protease like protein, defensin like protein*, and *C‐type lysozyme* were selected for RT‐qPCR after RPW larvae infected with EPF based on previous study (Abid Hussain et al. 2016). A total 1 µg of RNA was subjected to synthesize complementary DNA (cDNA) by reverse‐transcription (RT) by using the RT‐PCR MIX followed by user manual (Origin Pure BioSci & Tech.Co., TW). The RT reaction was performed at 42°C for 30 min and 80°C for 5 min. The qPCR reaction mixture consisted of 10 μL of iQ SYBR Green Supermix (BioRad), 1 μL of forward and reverse primers (10 μM), 1 μL of 1/10 × cDNA, and 7 μL of ddH_2_O. Primes sets used in RT‐qPCR are showed in Table S2. Thermal cycle of qPCR was consisted of a denaturation step at 95°C for 10 min, 40 cycles of 95°C for 15 s and 60°C for 60 s. The gene expressions were calculated by the $2^{‐△△C_{t}}$ method (Livak and Schmittgen 2001), the fold change of gene expressions were equals to log_2_2^‐△△Ct^.

### 2.8 16S Metagenomics Analysis

DNA samples from the EPF infected midgut and hindgut tissues were subjected to amplification of the V4‐V5 region of the 16S rRNA gene with the PCR primer set 515 f/806r, followed by DNA library construction and next‐generation sequencing (NGS). The sequencing library was sequenced using the Illumina MiSeq platform (Illumina, USA) to generate 2 × 300 bp paired‐end reads (BIOTOOLS, TW). The sequences obtained through NGS were presented in FASTQ file format and processed using Qiime2 v2023.5 for sequence analysis (Bolyen et al. 2019). Barcode sequences, PCR primers, and read with a median quality score below Q30 were filtered by the DADA2 plugin. Amplicon sequence variants (ASVs) were classified by using SILVA v138 classifier in Qiime2 (Quast et al. 2013). Family‐level classification was used in this study to demonstrate the composition of the gut microbiota. The alpha diversity of the gut microbiome within each sample was measured by Shannon diversity index (sampling depth = 16041). Boxplots of alpha diversity were generated using Qiime2 view. The beta diversity between different samples were assessed by Weighted‐UniFrac. Principle coordinates analysis (PCoA) plots were generated using EMPeror graphics tools in Qiime2.

### 2.9 Statistics Analysis

The significance of the differences in *B. bassiana* genome copy numbers between the fungal infected groups and the control groups was analyzed by Mann−Whitney *U* test. Spearman's correlation coefficient was used to calculate the correlation between gut microbiome alpha‐diversity, immunity‐related gene expression, and fungal genome copies. Specifically, Spearman's rank correlation analysis was performed to assess the relationship between the relative abundance of dominant bacterial families in digestive tissues (midgut and hindgut) and the expression levels of immune genes in the fat body To account for multiple testing, *p* values from Spearman correlations were adjusted using the Benjamini‐Hochberg method (Benjamini and Hochberg 1995), with significance thresholds set at a nominal *p* < 0.05 and a False Discovery Rate (FDR) < 0.25. The resulting correlation matrices were visualized using the package pheatmap (version 1.0.13) in R (version 4.5.1). General statistical analyses, including the Mann−Whitney *U* test and Spearman's correlation coefficient, were performed by IBM SPSS 20.

Differences in alpha‐diversity across groups or in pairwise comparisons were calculated using the Kruskal‐Wallis test followed by Dunn's test (Dunn 1964), while beta‐diversity differences were assessed by Permutational multivariate analysis of variance (PERMANOVA). Benjamini‐Hochberg method was used for multiple‐testing corrections after pairwise tests in both alpha‐diversity and beta‐diversity (Benjamini and Hochberg 1995). Benjamini‐Hochberg method was performed by package rstatix (version 0.7.3) for alpha‐diversity and the parameter p_adjust_method = “fdr” of package microeco (version 2.0.0) for beta‐diversity in R (version 4.5.1). The PERMANOVA were performed in Qiime2 (v2025.10). Differences in bacterial community composition were calculated by Linear discriminant analysis Effect Size (LEfSe) analysis, and visualized using Python version 3.8.20 (https://github.com/biobakery/biobakery/wiki/lefse). Unless otherwise specified, the general significance threshold was set at *p* < 0.05 for all statistical analyses.

## 3 Results

### 3.1 EPF Selection, Pathogenic Screening, and Bioassay Against RPW

**Figure 1:**
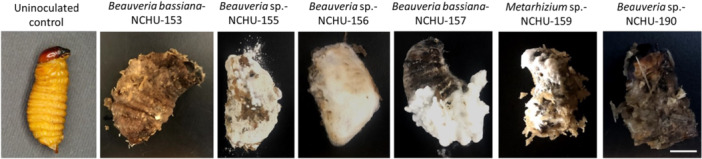
Observation of mycosis caused by six selected EPF isolates during pathogenicity screening. Scale bar = 1 cm.

A total of 13 fungal isolates were isolated from field‐collected RPWs (Table S4). Among these fungal isolates, five isolates, including NCHU‐153, 155, 156, 159, and 190 caused 100% mortality to 2nd mealworm pathogenicity screening at 4 dpi, and followed by the NCHU‐157, which showed mortality rate of 80% at 4 dpi (Table S4). Therefore, the six EPF isolates were subjected to pathogenicity screening against RPW. The infected RPW larva showed typical symptoms of fungal mycosis (Figure 1). Among the 6 isolates, the NCHU‐155 showed the highest mortality rate (100%) at 7 dpi, followed by NCHU‐153, 156 and 157 (Table 1). Therefore, NCHU‐155 was further selected to conduct the LT_50_ bioassay tests. The result of LT_50_ bioassay showed that the NCHU‐155 caused approximately 89% mortality in RPW larvae at 14 dpi with an LT_50_ value of approximately 8.7 days (Table 2).

**Table 1:** Pathogenicity screening of different isolates of EPFs against RPW larvae.

No.	Isolates No.	Species	7 dpi mortality rate (%)	14 dpi mortality rate (%)
1	Bb‐NCHU‐153	*Beauveria bassiana*	67	100
2	Bb‐NCHU‐155	*Beauveria* sp.	100	100
3	Bb‐NCHU‐156	*Beauveria* sp.	67	100
4	Bb‐NCHU‐157	*Beauveria bassiana*	67	67
5	Ma‐NCHU‐159	*Metarhizium* sp.	33	100
6	Bb‐NCHU‐190	*Beauveria* sp.	0	67

**Table 2:** The LT_50_ bioassay test of the Bb‐NCHU‐155 isolate against RPW larvae.

Replicates	14 dpi mortality rate (%)		Mean ± SD (%)		LT_50_ value of Bb‐NCHU‐155	*p* value
	Bb‐NCHU‐155	Control	Bb‐NCHU‐155	Control		
1st	73	7	89 ± 2.08	2 ± 3.30	8.7	0.001
2nd	93	0				
3rd	100	0				

### 3.2 Molecular Identification of EPF

**Figure 2:**
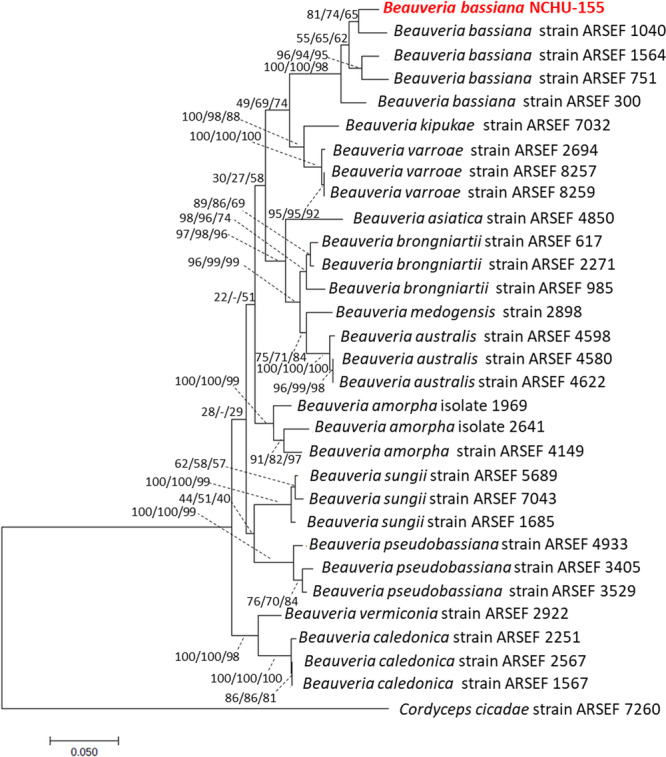
The phylogenetic analysis of *Beauveria bassiana* NCHU‐155. The tree was constructed by using the Tamura 3‐parameter model based on the intergenic regions B locus (*Bloc*) in MEGA7. Bootstrap analyses were performed to evaluate the robustness of the phylogenies using 1000 replicates. The values showing on each branch indicated the results evaluating from Minimum evolution/Neighbor‐Joining/Maximum Likelihood statistical methods and the tree pattern was presented according to the Maximum Likelihood method. “‐” means the value is under 50%.

Total eleven EPF isolates, including the NCHU‐153, 154, 155, 156, 157, 158, 159, 160, 188, 189, and 190, were subjected for molecular identification at the genus level based on the ITS region. The results revealed that the NCHU‐153, 155, 156, 157, 189, and 190 were belonged to the genus *Beauveria*, while the NCHU‐154, 158 and 159 were belonged to the genus *Metarhizium* (Table S4). Besides, NCHU‐160, and 188 were belonged to the genus *Metarhizium*, *Lecanicillium,* and *Meyerozyma*, respectively (Supplementary Table 4). Among these EPF isolates, the NCHU‐155 was selected for further confirmation at the species level through the phylogenetic analysis based on the intergenic region *bloc*. The results of phylogenetic analysis showed that the NCHU‐155 is an isolate of *B. bassiana* and is closely related to the *B. bassiana* strain ARSEF 1040, hereafter, the NCHU‐155 was named as *B. bassiana*‐NCHU‐155 (Bb‐NCHU‐155) (Figure 2).

### 3.3 *B. bassiana* Genome Copies in RPW Larvae After Infection

**Figure 3:**
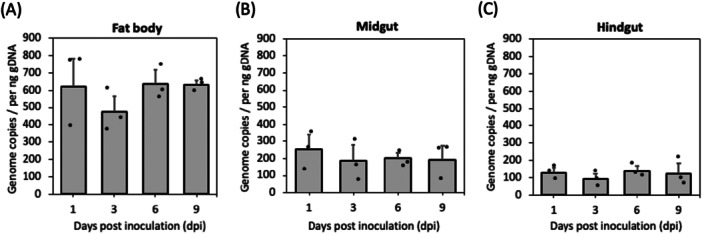
*Beauveria bassiana* genome copies in different tissues of EPF‐infected larvae. (A) Fat body, (B) Midgut and (C) Hindgut. Data are presented as mean ± SD (*n* = 3). Individual data points are overlaid on the bars.

Among these three tissues, the genome copies of Bb‐NCHU‐155 in the infected fat bodies were highest in the fat body, which ranged from 474.03 genome copies/per ng gDNA to 637.62 genome copies/per ng gDNA, followed by midgut (187.79 genome copies/per ng gDNA to 251.3 genome copies/per ng gDNA) and hindgut (91.83 genome copies/per ng gDNA to 138.24 genome copies/per ng gDNA) (Figure 3).

In the fat body, the genome copies of Bb‐NCHU‐155 showed an increasing tendency from 3 dpi to 6 and 9 dpi. (Figure 3). The results of genome copies quantification in the midgut showed a decreased trend from 1 dpi, while the genome copies of *B. bassiana* in the hindgut were lower than those of the midgut (Figure 3).

### 3.4 Immunity‐Related Gene Expression in *B. bassiana* Infected RPW Larvae

**Figure 4:**
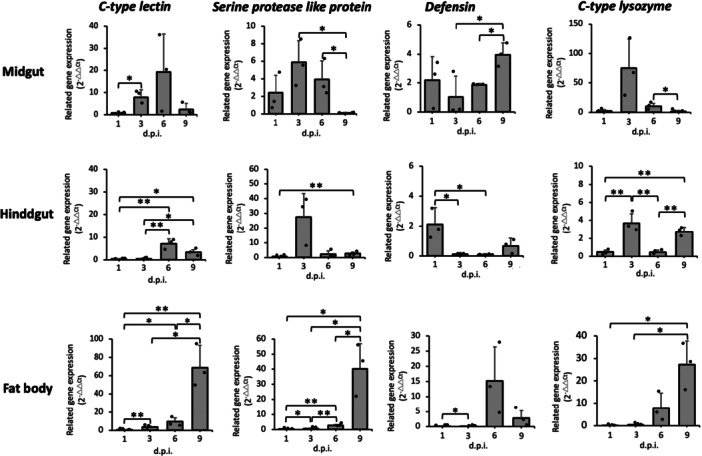
The related gene expression levels of four immunity‐related genes in the midguts, hindgut and fat body between EPF‐inoculated and non‐ inoculated RPW larvae at different time post‐inoculation were calculated by $2^{‐△△C_{t}}$ method. Data are presented as mean ± SD (*n* = 3) Individual data points are overlaid on the bars. * = *p* value < 0.05; ** = *p* value < 0.01.

In midgut tissue, the downregulation (0.78‐fold) of *C‐type lectin* was found at 1 dpi, while it showed significant upregulation at 3 dpi and reached the peak at 6 dpi (19.5‐fold) (Figure 4). The expression level of *serine protease like protein* and *C‐type lysozyme* exhibited upregulation pattern in midguts at 1 dpi, 3 dpi and 6 dpi, while the gene expression level significantly decreased at 9 dpi to 0.088‐fold and 1.75‐fold, respectively (Figure 4). For the *defensin*, the gene up expression was upregulated at 1 dpi. (2.1‐fold) and 9 dpi (3.9‐fold) in the midgut tissue, while downregulated at 3–6 dpi (Figure 4). It is worth noting that the highest expression level of *C‐type lysozyme* (74.7‐fold) was found in the midgut tissues at 3 dpi, indicating the tissue tropism of this gene in the midgut tissue.

In hindguts of Bb‐NCHU‐155 infected RPW larvae, *C‐type lectin*, *serine protease like protein* and *C‐type lysozyme* were downregulated at 1 dpi, while *defensin* was upregulated (2.1‐fold) at 1 dpi (Figure 4). Even though the *defensin* was upregulated at 1 dpi, the expression level of *defensin* was downregulated from 3 to 9 dpi in hindgut tissue (Figure 4). The expression level of *C‐type lectin* was turned into upregulation at 6 dpi (7.0‐fold) until 9 dpi (3.2‐fold), besides, *serine protease‐like protein* showed highest expression level (27.3‐fold) in hindgut at 3 dpi and continue to highly expressed at 9 dpi (2.7‐fold). The expression level of *C‐type lysozyme* also showed upregulation pattern at 3 (3.7‐fold) to 9 dpi (2.7‐fold) except 6 dpi exhibited a downregulation of gene expression (0.46‐fold) in hindgut tissue (Figure 4).

After Bb‐NCHU‐155 was infected with RPW larvae, the low expression levels of four immune‐related genes were found at 1 dpi to 3 dpi in fat bodies, while the expression levels of these genes were significantly upregulated from 1 dpi to 9 dpi, except *defensin* (downregulated at 9 dpi) (Figure 4). Besides, it was also noted that the highest gene expression level in fat bodies was *C‐type lectin* (68.4‐fold) and followed by *serine protease‐like protein* (40.1‐fold) *and C‐type lysozyme* (27.2‐fold) at 9 dpi, while the *defensin* showed highest expression level at 6 dpi (15.2‐fold) (Figure 4).

### 3.5 Sequence Summary and Gut Microbiome Composition

A total of 4,879,718 raw paired‐end (PE) reads of the 16S rRNA gene (V4‐V5 region) were obtained (Table S5). Low‐quality reads (Quality scores < 30) were removed by the DADA2 denoise‐paired and a total of 2,076,580 PE reads with lengths ranging from 274 to 464 bp were obtained for further analysis (Table S5). Based on the beta‐diversity results of RPW gut microbiome, the infected and control groups could be divided into different clusters (*p* = 0.012, PERMANOVA). Additionally, the clustering pattern showed that infection status (1–9 dpi) strongly influenced the gut microbial community structure (Figure S1A). Based on the alpha‐diversity results, the Shannon alpha‐diversity of the gut microbiome decreased over time in both the infection and control groups. Furthermore, the richness of the gut microbiome was generally lower in the infected group than in the control group, except 3 dpi, indicating that EPF infection may affect gut microbiome diversity (Figure S1B).

**Figure 5:**
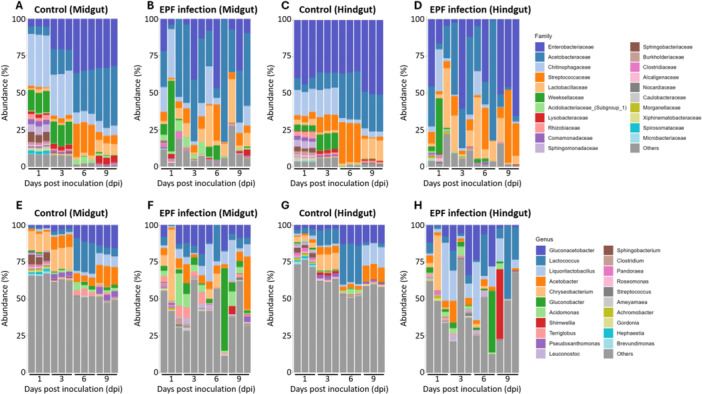
The sequence analysis of 16S V4‐V5 region from the midgut (A, B, E, and F) and hindgut (C, D, G, and H) tissues of non‐inoculated (A, C, E, and G) and EPF‐inoculated (B, D, F, and H) RPW larvae. Relative abundance of bacterial families (A−D) and genera (E ‐ H) in different treatments.

During 1‐9 dpi, Acetobacteraceae (midgut: 22.7%–51.5%, hindgut: 4.1%–48.6%) and Enterobacteriaceae (midgut: 8.5%–19.7%, hindgut: 12.9%–72.1%) were the major bacterial families in the gut of the infected group (Figure 5). In the midgut tissues, a similar bacterial composition was found at 1 dpi between control and infected RPW larvae, including Chitinophagaceae (control group = 10.9%; Infection group = 19.7%) and Weeksellaceae (control group = 18.9%; Infection group = 27.5%) as the top 3 bacterium (Figure 5). After 3 dpi, the group of Acetobacteraceae became the most abundant family, accounting for 51.5% of the community and remaining dominant until 9 dpi (47.3%) in the infected midgut (Figure 5). In the hindgut tissues, Enterobacteriaceae was the most abundant bacterial family from 1 to 9 dpi in the control group, while Acetobacteraceae became the dominant family in the infected RPW larvae at 3 dpi (47.2%) and 6 dpi (48.6%) (Figure 5). After 3 dpi, Lactobacillaceae and Streptococcaceae became the second most abundant bacterial families at 6 dpi and 9 dpi, respectively (Figure 5).

**Figure 6:**
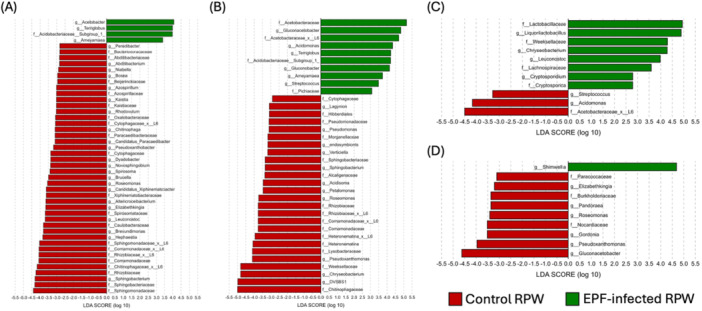
The Linear discriminant analysis Effect Size (LEfSe) analysis between non‐inoculated and EPF‐inoculated RPW larvae at (A) 1 dpi, (B) 3 dpi, (C) 6 dpi and (D) 9 dpi. Dpi = days post‐inoculation. The family and genus names of classified OTUs are prefixed with “f__” and “g__,” respectively.

LEfSe analysis revealed significant differences in the gut bacterial composition of EPF‐infected RPW larvae compared to control RPW larvae. At 1 dpi, the Acetobacteraceae family was enriched (Figure 6A). At 3 dpi, the Acetobacteraceae and Acidobacteriaceae families were enriched in the EPF‐infected RPW larvae (Figure 6B). At 6 dpi, Lactobacillaceae and Weeksellaceae were the predominant bacterial families in the gut of EPF‐infected RPW larvae (Figure 6C). At 9 dpi, Streptococcaceae was enriched in the gut of EPF‐infected RPW larvae (Figure 6D).

### 3.6 Correlation Between Gut Microbiota Dysbiosis and Systemic Immune Responses

**Figure 7:**
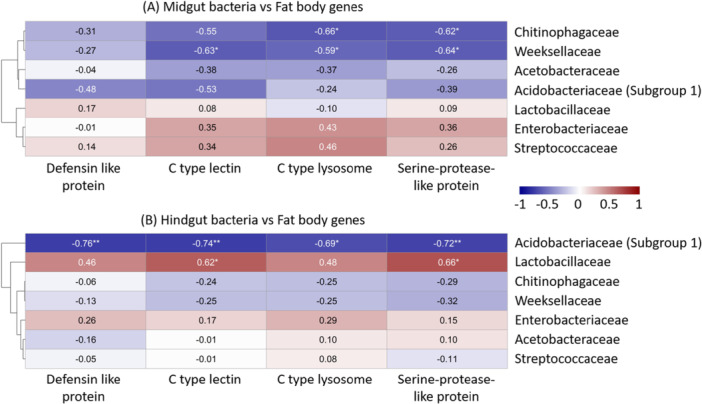
Spearman correlation analysis between gut bacterial abundance and immune gene expression in the fat body. Correlation heatmap between (A) midgut bacterial families and fat body immune gene expression, and (B) hindgut bacterial families and fat body immune gene expression. The color scale indicates the strength and direction of Spearman's rank correlation coefficient (Rho). * = *p* value < 0.05; ** = *p* value < 0.01.

To investigate whether the specific shifts in gut microbiota were associated with the host's systemic immune status, we analyzed the correlation between gut bacterial abundance and fat body immune gene expression (Figure 7). In the midgut (Figure 7A), the abundance of Lactobacillaceae and Enterobacteriaceae showed a strong positive correlation with the expression of *C‐type lectin*, *C‐type lysozyme*, and *serine protease‐like protein* in the fat body (Spearman's Rho > 0.8). This suggests that the bloom of these bacteria in the midgut coincides with the systemic activation of host immunity. Notably, in the hindgut (Figure 7B), the family Streptococcaceae exhibited a perfect positive correlation (Rho = 1.0) with the upregulation of *C‐type lectin*, *C‐type lysozyme*, and *serine protease‐like protein* in the fat body. Conversely, the abundance of Weeksellaceae and Chitinophagaceae in the hindgut showed a negative correlation with these immune markers. These results indicate a distinct, tissue‐specific pattern where specific hindgut taxa are highly synchronized with systemic immune interactions. Moreover, the immune‐related gene expression within digestive tissues was also examined. In the midgut, immune gene expression showed weaker correlations with resident microbiota compared to the systemic response (Figure S2A). However, in the hindgut, distinct local patterns were observed, where *Acidobacteriaceae* showed a strong negative correlation (Rho = −1.0) with local *C‐type lectin* expression (Figure S2B), further supporting the tissue‐specific immune regulation hypothesis.

## 4 Discussion

Preliminary pathogenicity screening clearly demonstrates that different isolates of the same EPF species exhibit varying degrees of virulence against RPW larvae (Table 1). Previous studies have reported that intra‐species variability among EPF isolates can result in significant differences in pathogenicity, potentially leading to host specificity (Lee et al. 2019; Yang et al. 2016). This variability is attributed to differences in enzymatic activity, specifically, the production of lipases, proteases, and chitinases involved in the penetration of the insect cuticle during infection (Golo et al. 2015). Such enzymatic diversity among isolates may explain the variation in host susceptibility and infection efficiency. Consistent with prior findings, RPW larvae exhibit differential susceptibility to various strains of *B. bassiana*, with certain isolates demonstrating superior infectivity (Abid Hussain et al. 2016; Uma Devi et al. 2008). Consequently, preliminary pathogenicity assessments are essential for identifying the most virulent and host‐compatible EPF isolates for potential use in biological control strategies against RPW infestations.

Following inoculation with Bb‐NCHU‐155, RPW larvae exhibited a mortality rate of 89% ± 2.08%, with a calculated median lethal time (LT_50_) of 8.70 days (Table 2). Although the LT_50_ was longer compared to other *B. bassiana* isolates such as JEF‐484 and JEF‐158 [6], Bb‐NCHU‐155 demonstrated a higher overall mortality rate. These results highlight the isolate's promise as a potent biocontrol agent against RPW. Nevertheless, the cryptic nature of RPW's life cycle and its concealed habitats present significant challenges for effective field‐level application of EPF. Therefore, further field trials are imperative to evaluate the practical efficacy, environmental persistence, and formulation stability of Bb‐NCHU‐155 under natural conditions.

Another concern is that the larval developmental stage is a critical factor influencing EPF pathogenicity. Larger and more mature RPW larvae generally exhibit higher LC_50_ values, indicating increased resistance to fungal infection relative to younger instars (Aldossary et al. 2009; Sabbour and Abdel‐Raheem 2014).

In the current study, late‐instar larvae (7th to 8th instar larvae) were selected for bioassay with Bb‐NCHU‐155. The genome copies of Bb‐NCHU‐155 of both midgut and hindgut were lower than those of fat body, indicating the order of the fungal infection process is mainly from cuticle into the hemolymph and fat body, while the midgut and hindgut might not be the main tissues for fungal infection (Figure 3). Compared to the initial inoculation (10^7^ conidia/mL), the replication of *B. bassiana* was suppressed in all three tissues during the infection process. Therefore, it was worthy to investigate the host response against EPF infection based on the immunity‐related gene expression patterns to clarify this hypothesis.

The expression of four immunity‐related genes in gut and fat bodies of infected RPW larvae was detected. The four immune‐related genes in the fat bodies exhibited downregulation at 1 dpi (Figure 4). Previous studies have reported that EPF could secrete bioactive compounds which suppress the host immune responses. *M. destruxins* are capable of inhibiting the expression of genes encoding AMPs and blocking phagocytosis by inhibiting V‐ATPase (Chen et al. 2014). Similarly, *B. bassiana* produces bibenzoquinone oosporein, which inhibit ProPO activities and downregulates the expression of gallerimycin in wax moth, *Galleria mellonella*, (L.) (Feng et al. 2015), and the AMPs gene expression in mosquito midguts (Wei et al. 2017). However, a gradually increasing of these four immune‐related genes in the fat bodies from 3 dpi, indicating the typical host response to the fungal infection (Figure 4). Indeed, it was known that fat body is a major immune responsive organ in insects (Charroux and Royet 2010; Li et al. 2019). The research result of Abid Hussain et al. (2016) showed that *C‐type lectin*, *serine protease like protein*, *defensin*, and *C‐type lysozyme* were expressed highly after the infection of *B. bassiana*. It can be suggested that the defensin‐like protein in the fat body could involve in immune response and suppressed in the duplication of *B. bassiana*.

In the digestive system, the immunity‐related genes revealed a fluctuating pattern. In the midgut, the *C‐type lysosome* showed the highest expression level (~74.7‐fold) at 3 dpi. and followed by *C‐type lectin* (19.3‐fold) at 6 dpi; in the hindgut tissue, the highest gene expression level (27.3‐fold) of serine protease‐like protein was detected at 3 dpi, while a downregulation trend of four immune‐related genes was also observed in the hindguts, indicating that the infection of EPF may trigger the specific immune‐genes in different tissues (Figure 4). It was reported that *B. bassiana* can interact with gut bacteria to downregulate the expression of AMPs and dual oxidase in mosquito midguts (Wei et al. 2017). Based on the infection process of *B. bassiana* detected in the midguts and hindguts, *B. bassiana* may not have infected the midguts at 1 dpi. The downregulation of immune‐related genes in hindguts may be attributed to the inhibitory effects of *B. bassiana*.

In examining the gut microbiome of EPF‐infected RPW larvae, notable compositional changes were observed following infection (Figure 5). LEfSe analysis revealed that members of the family Acetobacteraceae were significantly enriched at 1 and 3 dpi (Figure 6). Several genera within Acetobacteraceae are classified as acetic acid bacteria (AAB) (Crotti et al. 2010), which are known gut symbionts implicated in modulating host immunity in fruit flies (*Drosophila* spp.). For instance, Ryu et al. (2008) demonstrated that the gut microbiota of healthy fruit flies is dominated by two AAB strains‐*Acetobacter pomorum* and *Commensalibacter intestine*‐which suppress the growth of the pathogenic bacterium *Gluconobacter morbifer* through competitive exclusion. In addition, the symbiotic bacterium Acidomonas, found on sugarcane, has demonstrated resistance against pathogenic fungi *Fusarium moniliforme* (Pitiwittayakul et al. 2021). The enrichment of AAB in the gut of EPF‐infected RPW larvae may thus play a role in suppressing pathogenic bacteria and fungal infections, potentially through immune modulation or microbial competition.

At 6 dpi, the gut microbiome was dominated by bacterial families Lactobacillaceae and Weeksellaceae (Figure 6). Members of the genus *Lactobacillus*, within Lactobacillaceae, have been shown to modulate the immune system in *G. mellonella*, thereby preventing fungal infections (Rossoni et al. 2017). Conversely, some genera within Weeksellaceae have been identified as pathogens in other insects, such as the western corn rootworm (*Diabrotica virgifera virgifera*) and the soft tick (*Ornithodoros moubata*) (Burešová et al. 2006; Jabeur et al. 2023). This suggests that Weeksellaceae may also act as a potential pathogen in RPW larvae.

By 9 dpi, members of the family Streptococcaceae were enriched in the gut microbiome of infected larvae (Figure 6). The genus *Lactococcus*, within this family, has demonstrated antifungal activity against *Colletotrichum capsici*, the causal agent of anthracnose in chili peppers (Fakri et al. 2022). Additionally, *Lactococcus* is believed to enhance resistance to pathogens in insects by promoting gut acidification (Tuerlings et al. 2023). Therefore, the increased abundance of Streptococcaceae may contribute to fungal suppression in EPF‐infected RPW larvae.

While alpha diversity of the gut microbiota showed a slight, non‐significant increase following EPF infection, beta diversity differed significantly between infected and control larvae, indicating distinct microbial community structures (Figure S1). Previous studies have shown that pathogen infections can alter gut microbiota in insects (Broderick et al. 2009; Jakubowska et al. 2013; Wang et al. 2022). However, to date, no studies have specifically reported shifts in the RPW gut microbiome in response to pathogenic infection. Our findings demonstrate that EPF infection indeed induces changes in the gut microbiota of RPW larvae.

Moreover, our results suggest a possible association between increased gut microbial alpha diversity and elevated expression of immunity‐related genes (Figure 7). The gut microbiota may play a role in stimulating immune responses in RPW larvae (Muhammad et al. 2019), supporting the hypothesis that the microbiome contributes to host defense against EPF. Notably, Weeksellaceae, considered a bacterial pathogen in insects, was positively correlated with the expression of *C‐type lectin*, implying it may trigger immune responses (Figure 7). Similarly, Chitinophagaceae has been reported to correlate positively with immune gene expression (Fuess et al. 2021). However, the relationship between Acidobacteriaceae and host immunity remains unexplored. Further research is needed to elucidate the functional roles of specific microbial taxa and their interactions with immunity‐related gene expression in RPW larvae.

## 5 Conclusion

In conclusion, this study demonstrates that infection by Bb‐NCHU‐155 induces immune gene expression, particularly in the fat body, and alters the gut microbiota composition in RPW larvae. Among the immune responses, defensin appears to play a key role in suppressing fungal infections within the fat body tissue. Our findings reveal significant correlations between specific gut microbial taxa and immunity‐related gene expression, underscoring the complex interplay between host immunity and gut microbial dynamics. These insights provide valuable knowledge for developing microbiome‐informed biocontrol strategies against RPW. However, to fully understand the mechanisms driving these interactions, further studies are needed to characterize the functional roles of specific microbial taxa and their direct contributions to immune regulation. The development of agents that disrupt beneficial gut bacteria in RPW, when used in combination with EPF, may enhance the efficacy of biological control approaches. Nevertheless, this hypothesis requires further experimental validation before practical application.

## Author Contributions

**Tzu‐Hao Yang:** investigation, methodology, writing – original draft. **Fang‐Min Chang:** data curation, validation, writing – original draft, writing – review and editing. **Pin‐Chang Chen:** methodology, validation, visualization. **Rameshwor Pudasaini:** data curation, validation, writing – original draft, writing – review and editing. **Hsiao‐Pei Lu:** conceptualization, supervision, resources, writing – original draft, writing – review and editing. **Yu‐Shin Nai:** conceptualization, supervision, resources, writing – review and editing, writing – original draft.

## Conflicts of Interest

The authors declare no conflicts of interest.

## Supporting information


**Figure S1:** The sequence analysis of 16S V4‐V5 region from the midgut and hindgut tissues of EPF‐infected and control RPW larvae. (A) The weighted‐unifrac beta‐diversity of RPW gut microbiome. (B) The statistics of beta‐diversity of RPW gut microbiome calculated using PERMANOVA. The bold italic values in the upper right blocks represent p‐values, while the values in the lower left blocks represent *q*‐values. *p‐v*alues less than 0.05 are shown in green, and *p*‐values greater than 0.05 are shown in red. (C) Boxplot of EPF‐infected and control RPW larvae gut microbiome Shannon entropy alpha‐diversity, differences in alpha‐diversity across groups or in pairwise comparisons were labeled by lowercase letters.

**Figure S2:** Spearman correlation analysis of local microbiota‐immune interactions. Correlation heatmap between (A) midgut bacterial families and midgut immune gene expression; and (B) hindgut bacterial families and hindgut immune gene expression. The color scale indicates the strength of the Spearman's rank correlation coefficient (Rho). Compared to the systemic interactions (Figure 6), local interactions in the midgut were less synchronized, while the hindgut showed specific positive (Rho > 0.5 and p‐value < 0.05) correlations between *Enterobacteriaceae* and antimicrobial peptides, reflecting a complex local immune tolerance environment. *= *p*‐value < 0.05.

**Table S1:** The information of palm tree fields for RPW collection in Taiwan. **Table S2:** Primer sets used in this study. **Table S3:** The GenBank accession number of bloc used for phylogenetic analysis. **Table S4:** Isolation and selection of entomopathogenic fungi (EPF) from red palm weevil, RPW (*Rhynchophorus ferrugineus*) by mealworm (*Tenebrio molitor*) test. **Table S5:** Sequencing summary. **Table S6:** Summary of Spearman's rank correlation coefficient analysis.

## Data Availability

The data that support the findings of this study are available in NCBI at https://www.ncbi.nlm.nih.gov/bioproject/PRJNA1227604/, reference number PRJNA1227604. These data were derived from the following resources available in the public domain: —SRA Experiments, https://www.ncbi.nlm.nih.gov/sra?linkname=bioproject_sra_all&from_uid=1227604. The GenBank accession numbers of Bb‐NCHU‐155 identified in this study, which were used for phylogenetic analysis, are listed in Table S3. The NGS sequencing data have been deposited in NCBI under BioProject accession number PRJNA1227604.
