# Supplementary material for: Immune Response and Gut Microbiota Shift in the Red Palm Weevil (Rhynchophorus ferrugineus) Infected With Entomopathogenic Fungus, Beauveria bassiana, Reveal Host‐Pathogen Interactions
Source: Arch Insect Biochem Physiol. 2026 Jun 23;122(2):e70183. doi: 10.1002/arch.70183 (PMC13290068; doi:10.1002/arch.70183)

(A)

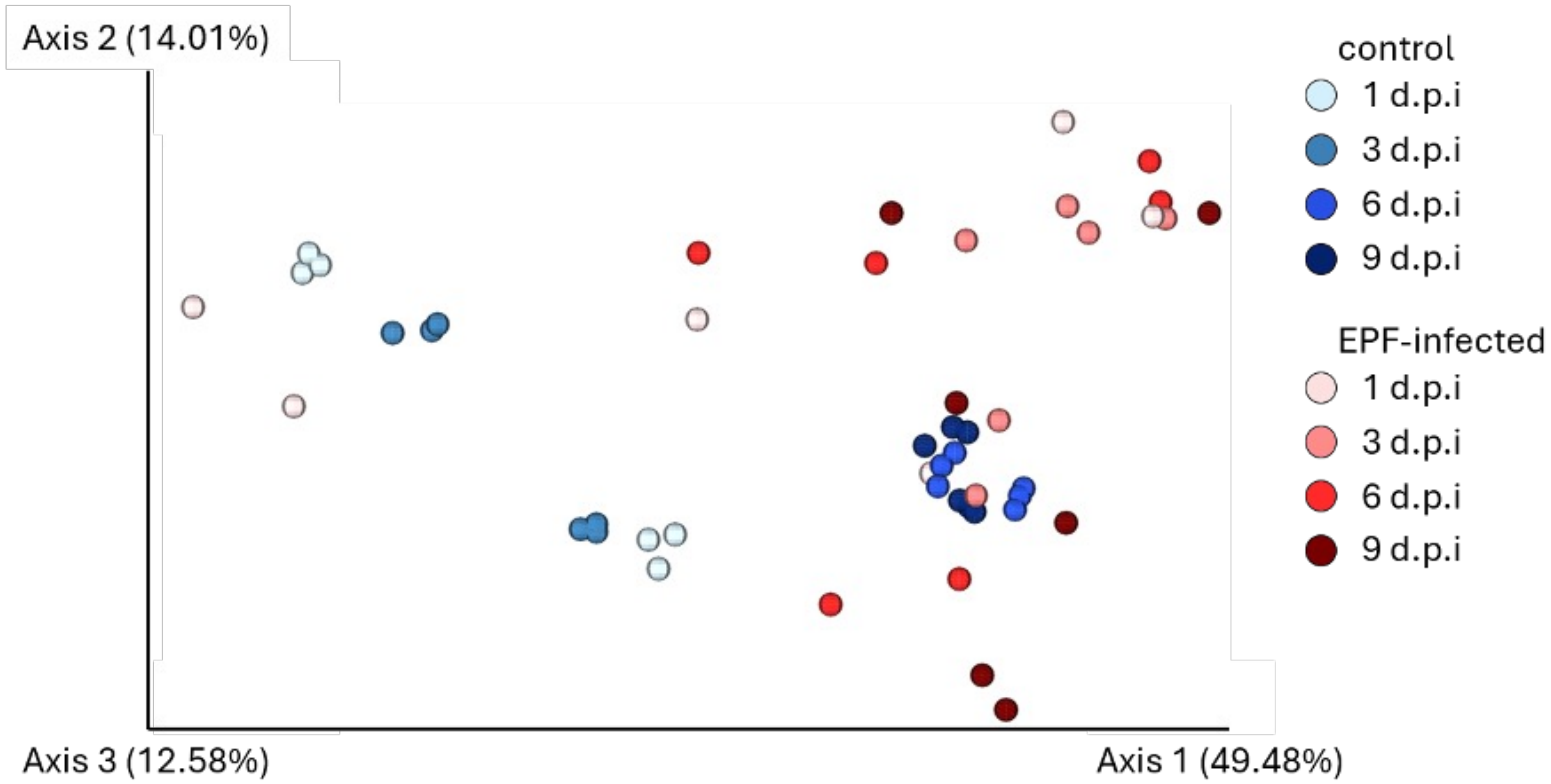

(B)

		Control				EPF-infected			
		1 d.p.i	3 d.p.i	6 d.p.i	9 d.p.i	1 d.p.i	3 d.p.i	6 d.p.i	9 d.p.i
Control	1 d.p.i	-	***0.001***	***0.002***	***0.002***	***0.003***	-	-	-
	3 d.p.i	0.0047	-	***0.003***	***0.001***	-	***0.008***	-	-
	6 d.p.i	0.0047	0.0053	-	***0.008***	-	-	***0.003***	-
	9 d.p.i	0.0047	0.0047	0.0102	-	-	-	-	***0.01***
EPF-infected	1 d.p.i	0.0053	-	-	-	-	***0.255***	***0.001***	***0.113***
	3 d.p.i	-	0.0102	-	-	0.255	-	***0.004***	***0.186***
	6 d.p.i	-	-	0.0053	-	0.0047	0.0059	-	***0.002***
	9 d.p.i	-	-	-	0.0122	0.1217	0.1929	0.0047	-

(C)

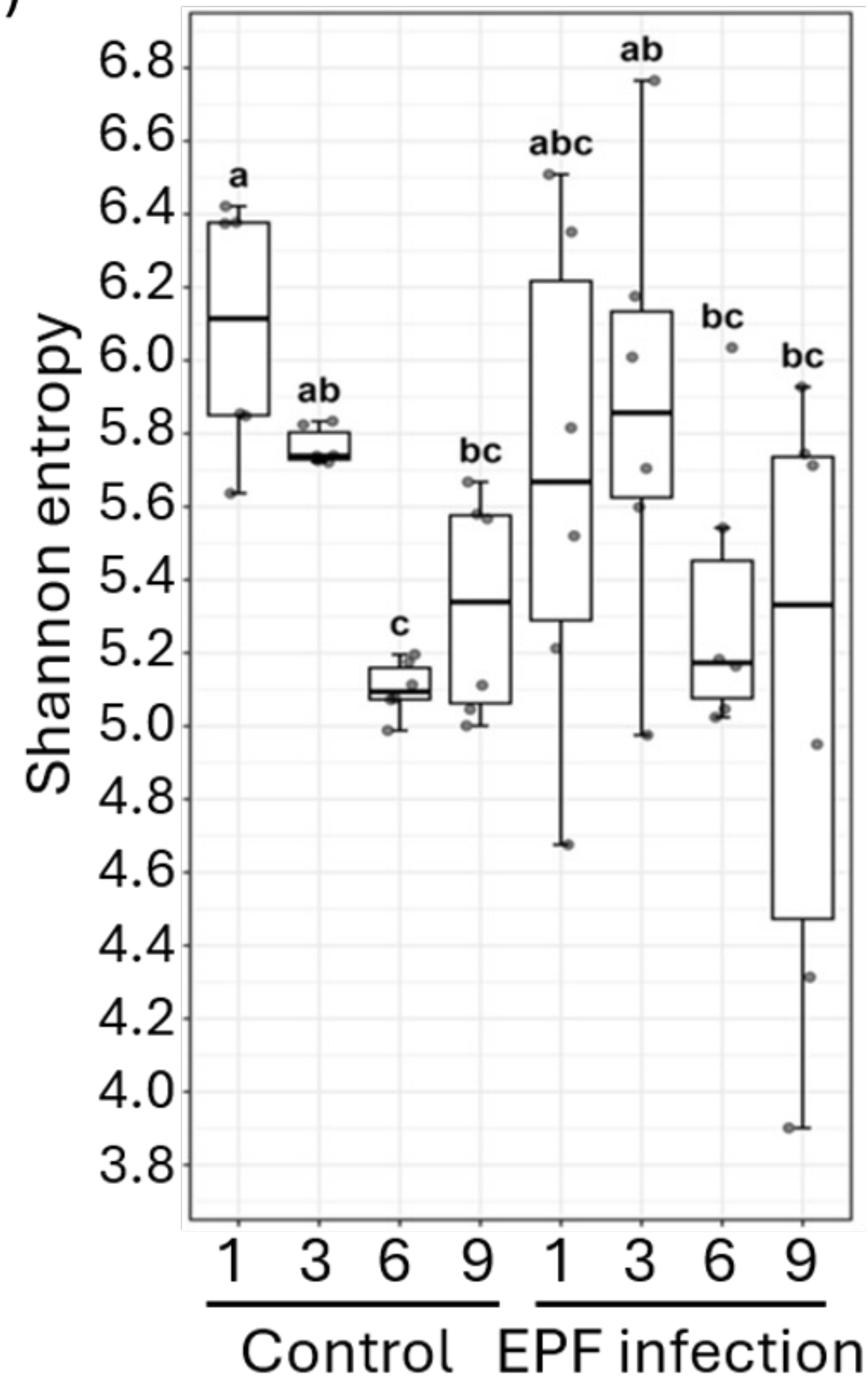

Supplement: Supplementary file 2 — Figure S2: Spearman correlation analysis of local microbiota‐immune interactions. Correlation heatmap between (A) midgut bacterial families and midgut immune gene expression; and (B) hindgut bacterial families and hindgut immune gene expression. The color scale indicates the strength of the Spearman's rank correlation coefficient (Rho). Compared to the systemic interactions (Figure 6), local interactions in the midgut were less synchronized, while the hindgut showed specific positive (Rho > 0.5 and p‐value < 0.05) correlations between Enterobacteriaceae and antimicrobial peptides, reflecting a complex local immune tolerance environment. *= p‐value < 0.05. [file ARCH-122-e70183-s001.pdf]
