# Supplementary figures and images for: Immune Response and Gut Microbiota Shift in the Red Palm Weevil (Rhynchophorus ferrugineus) Infected With Entomopathogenic Fungus, Beauveria bassiana, Reveal Host‐Pathogen Interactions
Source: Arch Insect Biochem Physiol. 2026 Jun 23;122(2):e70183. doi: 10.1002/arch.70183 (PMC13290068; doi:10.1002/arch.70183)

(A) Midgut bacteria vs Midgut genes

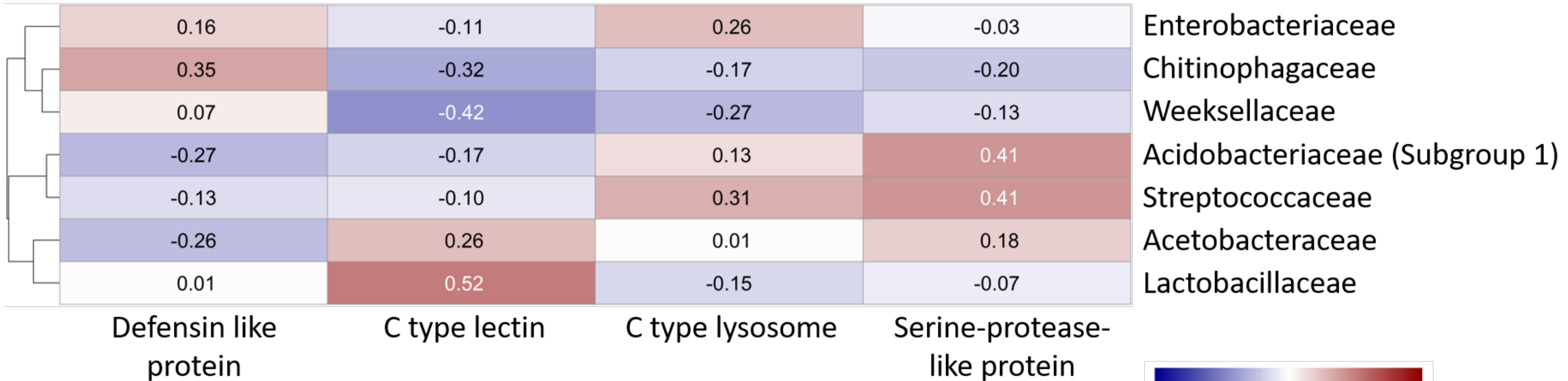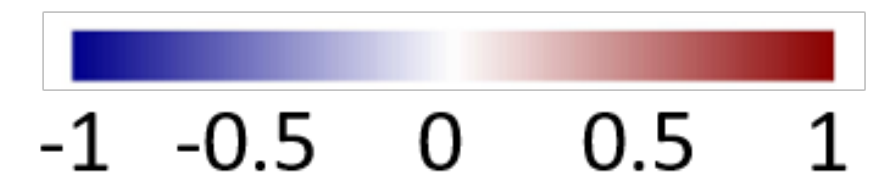

(B) Hindgut bacteria vs Hindgut genes

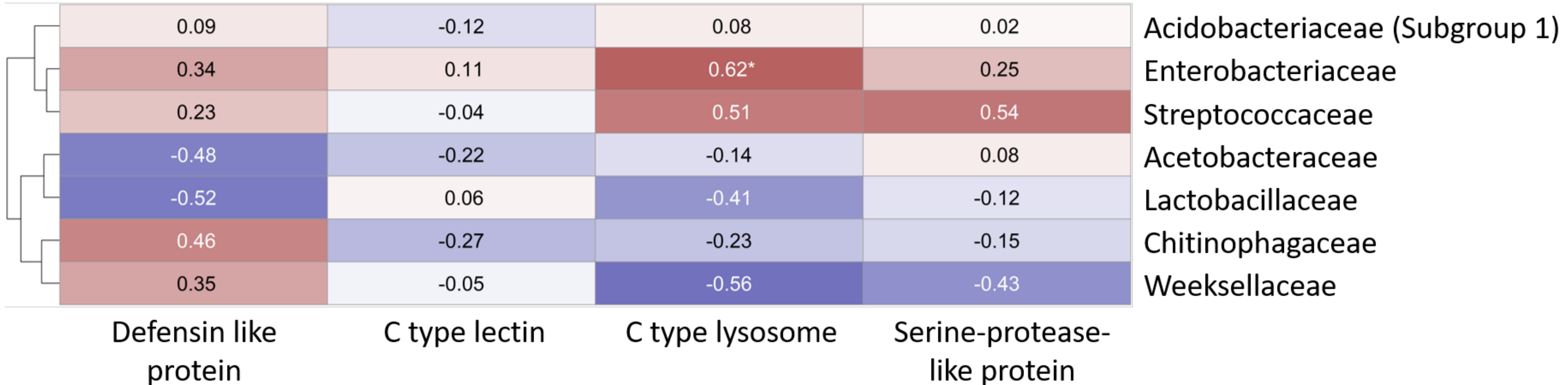

Supplement: Supplementary file 3 — Table S1: The information of palm tree fields for RPW collection in Taiwan. Table S2: Primer sets used in this study. Table S3: The GenBank accession number of bloc used for phylogenetic analysis. Table S4: Isolation and selection of entomopathogenic fungi (EPF) from red palm weevil, RPW (Rhynchophorus ferrugineus) by mealworm (Tenebrio molitor) test. Table S5: Sequencing summary. Table S6: Summary of Spearman's rank correlation coefficient analysis. [file ARCH-122-e70183-s002.pdf]
